# Features of patients with advanced EGFR-mutated non-small cell lung cancer benefiting from immune checkpoint inhibitors

**DOI:** 10.3389/fimmu.2022.931718

**Published:** 2022-08-05

**Authors:** Qian Chen, Xiaoling Shang, Ni Liu, Xinchun Ma, Wenfei Han, Xiuwen Wang, Yanguo Liu

**Affiliations:** Department of Medical Oncology, Qilu Hospital of Shandong University, Jinan, China

**Keywords:** non-small cell lung cancer, EGFR mutation, immune checkpoint inhibitors, PD-L1 expression, treatment paradigm

## Abstract

**Background:**

Although immune checkpoint inhibitors (ICIs) generally show poor therapeutic efficacy in patients with epidermal growth factor receptor (EGFR) mutations, certain research indicate that a small proportion of these patients do respond to ICIs. The present study sought to identify the features of patients with EGFR mutations who might benefit from ICIs from multiple studies and discussed the optimal treatment paradigm for advanced non-small cell lung cancer (NSCLC) patients with EGFR mutations.

**Methods:**

The profiles of 114 advanced NSCLC patients with EGFR mutations who received ICIs treatment were retrospectively reviewed. EGFR subtypes, programmed cell death ligand 1 (PD-L1) expression, and clinical characteristics regarding their impact on the efficacy of ICIs were investigated.

**Results:**

Patients with major EGFR mutations (L858R or 19Del) had a shorter progression-free survival (PFS) and a lower objective response rate (ORR) as compared to patients with rare (20ins or G719X) and other EGFR mutations. Although not statistically significant, median overall survival (OS) tended to be longer in patients with negative (<1%) PD-L1 expression than with positive (≥1%) PD-L1 expression (15.61 vs. 7.40 months, p = 0.138). Median PFS and OS were significantly shorter in heavily treated patients (prior lines of therapy ≥3 lines vs. <3 lines: mPFS, 1.80 vs. 2.50 months, p = 0.003; mOS, 6.70 vs. 14.00 months, p = 0.031). ORR was also lower in patients who had received ≥3 prior lines of therapy compared to in those <3 prior lines of therapy (0.00% vs. 21.67%, p = 0.002).

**Conclusion:**

Patients with major EGFR mutations showed poorer responses to ICIs than those with rare EGFR mutations. EGFR-mutated patients with lower PD-L1 expression showed a trend towards a longer OS after receiving ICIs. ICIs should be administered as early as possible to previously treated EGFR-mutated NSCLC patients. ICI-based combined therapies may be a direction for treatment of these patient subtypes in the future.

## Introduction

According to GLOBOCAN 2020, lung cancer is the second most frequently diagnosed cancer worldwide and remains the leading cause of cancer deaths (1). In China, lung cancer remains the most common type of cancer, being responsible for 0.72 million deaths in 2020 (2). Epidermal growth factor receptor (EGFR) gene mutations are the most common driver gene alterations in Asian patients with lung adenocarcinoma, with an overall mutation frequency of 51.4% (3). EGFR-tyrosine kinase inhibitors (TKIs) have dramatically improved the survival of patients harboring EGFR mutations. For example, the first-generation EGFR-TKIs gefitinib was shown to significantly improve 12-month rates of progression-free survival (PFS) compared with platinum-containing chemotherapy (24.9% vs. 6.7%) in the Iressa Pan-Asia Study (IPASS) (4). Second-generation EGFR-TKIs, such as afatinib and dacomitinib, not only showed longer PFS and a higher objective response rate (ORR) than chemotherapy but also had better therapeutic efficacy in patients with rare EGFR mutations (such as G719X and L861Q) (5). First- and second-generation TKIs showed a median PFS (mPFS) of 9–13 months. As for third-generation TKIs, the FLAURA study revealed that osimertinib increased patients’ mPFS to 18.9 months (6). In addition to the success of EGFR-TKI monotherapy, combined treatments including these agents have also demonstrated survival improvements (7). Consequently, EGFR-TKIs are the recommended first-line standard treatment for EGFR-mutated NSCLC. However, almost all patients will develop resistance to EGFR-TKIs, eventually leading to disease progression (8). It is therefore of great importance to seek further treatment strategies.

Compared with EGFR-TKIs, immune checkpoint inhibitors (ICIs) have shown promising antitumor effects in non-small cell lung cancer (NSCLC) and are considered to have long-term responses that can potentially lead to cure (9–13). Unfortunately, ICIs showed limited benefits in EGFR-mutated patients compared to those who were EGFR wild type (14, 15). However, the ATLANTIC trial, a phase II single-arm study, showed that EGFR-mutant patients with ≥25% programmed cell death ligand 1 (PD-L1) expression had higher ORR and encouraging medium overall survival (mOS) when treated with durvalumab, a PD-L1 inhibitor, suggesting that a subgroup of patients with EGFR mutations may benefit from ICIs (16, 17). Few studies to date have assessed the efficacy of ICIs in patients with EGFR mutations, especially the application of ICIs alone [anti-PD-1 or anti-PD-L1, with or without cytotoxic T-lymphocyte-associated protein 4 (CTLA-4) blockade]. Most clinical studies on ICIs in patients harboring EGFR mutations are ICI-based combined therapies (with chemotherapy or chemotherapy plus anti-angiogenic therapy).

The present study was designed to investigate the efficacy of ICIs in patients with EGFR-mutated NSCLC. Our study, with a relatively large sample size, collected 114 patients with EGFR-mutated NSCLC who were treated with ICIs alone from three previous studies. Analysis of this patient population may better clarify the characteristics of NSCLC patients with EGFR mutations who could benefit from ICIs.

## Materials and methods

### Study design and patient population

**Figure 1:**
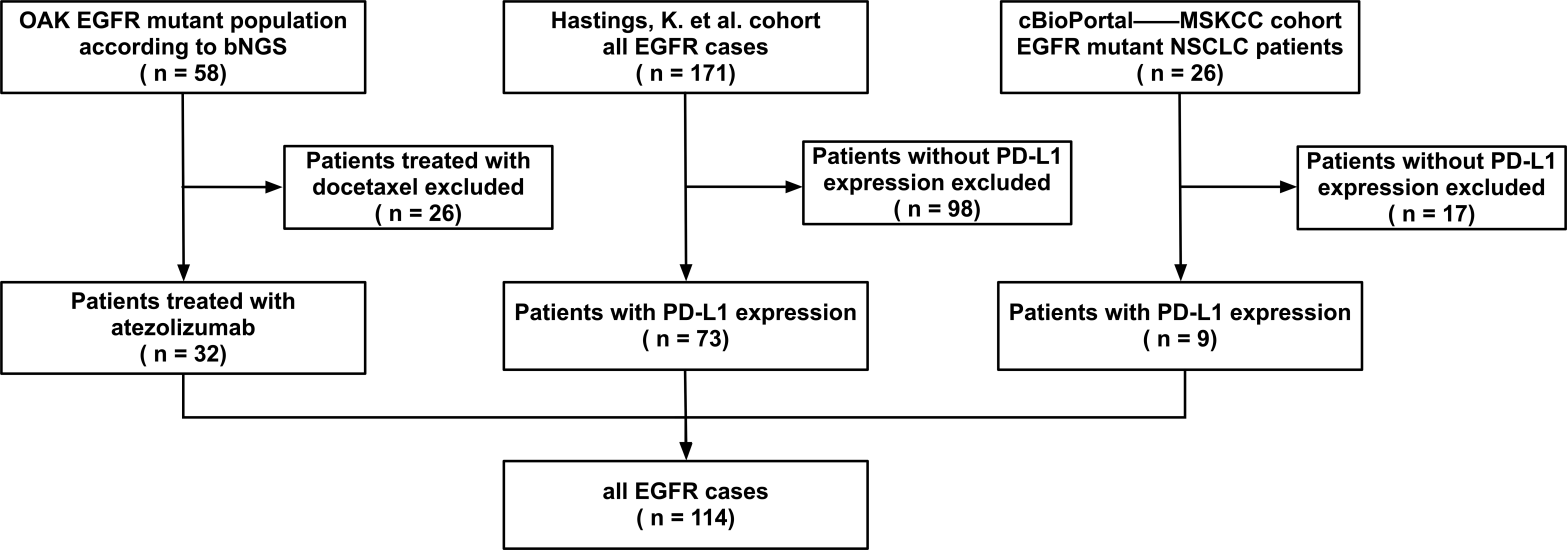
Patient selection. Epidermal growth factor receptor (EGFR) mutated non-small cell lung cancer patients treated with immune checkpoint inhibitors from OAK, Memorial Sloan Kettering Cancer Center (MSKCC), and Hastings et al. cohorts were selected (n = 229). A total of 114 patients who had the information of programmed cell death ligand 1 (PD-L1) expression were included.

In our study, data from EGFR-mutated NSCLC patients receiving ICIs (anti-PD-1 or anti-PD-L1, with or without anti-CTLA-4) were collected, and 114 patients with PD-L1 expression were finally identified from three studies. The flow chart in **Figure 1** shows the details of patient collection. Data from the OAK trials were obtained from the original study (18). Data on patients treated at Memorial Sloan Kettering Cancer Center (MSKCC) were downloaded from cBioPortal (19). Data on NSCLC patients with EGFR mutations treated with PD-(L)1 blockade therapy at Yale Cancer Center, MSKCC, the University of California Los Angeles, and Dana Farber Cancer Institute were obtained from the original study by Hastings et al. (20). Major EGFR mutations included L858R and exon 19 deletions (19Del), whereas rare EGFR mutations included exon 20 insertions (20ins) and G719X. Patients with both major and rare or other EGFR mutations were classified as having major EGFR mutations.

### Study outcomes

The outcomes included PFS, overall survival (OS) and ORR. The definition of PFS and OS among the three included studies were consistent. PFS was defined as the time from the initiation of ICIs treatment to the day of disease progression or death from any cause, and OS was defined as the time from the initiation of ICIs treatment to death from any cause. ORR was defined as the proportion of patients who achieved complete response (CR) and partial response (PR). CR and PR were assessed according to Response Evaluation Criteria in Solid Tumors (RECIST) version 1.1 criteria in all three included studies.

### Statistical analysis

PFS and OS were visualized using Kaplan–Meier curves, and differences between groups were analyzed using the log-rank test. ORR across different groups were compared using Fisher’s exact tests. Chi-square tests were utilized to compare clinical variables in patients subcategorized by their PD-L1 expression levels. Log-rank test and Cox regression for univariate and multivariate analyses were used to assess risk factors for PFS in 114 EGFR-mutated patients receiving ICIs. Statistical analyses were performed using the SPSS 26.0 software package and GraphPad Prism 9.0.0, with a two-sided p-value < 0.05 considered statistically significant.

## Results

### Clinical characteristics of NSCLC patients with EGFR mutations

A retrospective review of patients in the three studies identified 229 EGFR-mutated patients who were treated with ICIs. PD-L1 expression was assessed in 114 of these patients; their demographic and clinical characteristics are shown in **Table 1**. Of these 114 patients, 56 (49.1%) were smokers, and 109 (95.6%) had received previous treatment before ICIs, with only five (4.4%) being treatment naive. Fifty-seven (50.0%) patients were positive for PD-L1 expression (≥1%), including 14 (12.3%) with high (≥50%) PD-L1 expression.

**Table 1:** Patients’ characteristics.

Characteristics	All patients (n = 114)	
	Number (n)	Percent (%)
**Gender**		
Female	74	64.91
Male	40	35.09
**Age**		
Age<65	75	65.79
65≤Age<75	28	24.56
Age≥75	11	9.65
**Smoking history**		
Ever	56	49.12
Never	58	50.88
**Prior lines of therapy**		
0	5	4.39
1–2	68	59.65
≥3	41	35.96
**Treatment**		
Monotherapy	100	87.72
Combination	14	12.28
**PD-L1**		
<1%	57	50.00
1–49%	43	37.72
≥50%	14	12.28
**EGFR status**		
Major	70	61.40
Rare	22	19.30
Others	14	12.28
T790M *	6	5.26
NA	2	1.75
**T790M status**		
Positive	35	30.70
Negative	76	66.67
NA	3	2.63

*Primary T790M mutation.

### Associations between the efficacy of ICIs and EGFR subtypes

**Figure 2:**
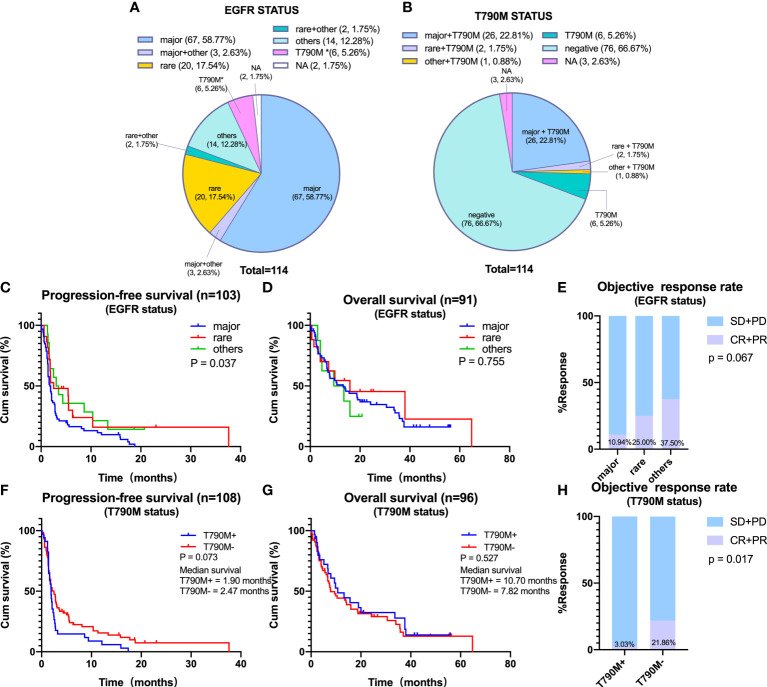
Efficacy of immune checkpoint inhibitors on distinct epidermal growth factor receptor (EGFR) subtypes. **(A)** Percentage of patients with non-small cell lung cancer containing major, rare, and other EGFR mutations. Major EGFR mutations include L858R and 19Del, and rare mutations include 20ins and G719X. **(B)** Percentage of patients according to T790M status. **(C–E)** Progression-free survival (PFS) **(C)**, overall survival (OS) **(D)**, and objective response rate (ORR) **(E)** in patients harboring major, rare, or other EGFR mutations. **(F–H)** PFS **(F)**, OS **(G)**, and ORR **(H)** in patients harboring EGFR T790M or negative for EGFR T790M. *Primary T790M mutation.

The associations of EGFR and T790M status with the efficacy of ICIs was investigated. Of the 114 included patients, 70 (61.4%) had major EGFR mutations; 22 (19.3%) had rare EGFR mutations, including 13 (11.4%) with 20ins and 9 (7.9%) with G719X mutations, and 14 (12.3%) had other mutations of unknown significance (**Figure 2A**). In addition, 35 (30.7%) patients were T90M positive (**Figure 2B**).

The associations between the efficacy of ICIs and the types of EGFR mutations were assessed by determining PFS, OS, and ORR in NSCLC patients with major, rare, or other EGFR mutations. Median PFS was significantly shorter in patients with major than with rare or other EGFR mutations (major vs. rare vs. others: mPFS, 1.82 vs. 2.50 vs. 3.26 months, p = 0.037) (**Figure 2C**). However, median OS did not differ significantly in these three subgroups (**Figure 2D**). In addition, ORR tended to be lower in patients with major than with rare or other EGFR mutations (10.94% vs. 25.00% vs. 37.50%, p = 0.067) (**Figure 2E**). In comparison of patients with major EGFR mutations, L858R and 19Del showed no statistically significant differences in PFS, OS, and ORR (**Supplementary Figures 1A–C**).

Furthermore, we analyzed the efficacy of ICIs according to T790M status. Results showed that neither PFS nor OS in response to ICIs treatment differed significantly between T790M-positive and T790M-negative patients, although ORR was significantly higher in T790M-negative than in T790M-positive patients (21.86% vs. 3.03%, p = 0.017) (**Figures 2F–H**).

### Relationship between PD-L1 expression and the efficacy of ICIs

PD-L1 expression was the most widely used biomarker that was closely related to efficacy of ICIs, especially for advanced NSCLC patients with EGFR wild type. Therefore, the relationship between the expression of PD-L1 and the efficacy of ICIs in EGFR-mutated patients was evaluated. Gender, age, and smoking history did not differ in patients with tumors negative (<1%), medium (1–49%), and high (≥50%) for PD-L1 expression, with EGFR status and T790M status also being similar in these patient subgroups (all p > 0.05, **Table 2**).

**Table 2:** Risk factors analysis of PD-L1 expression in EGFR-mutated patients.

Variables	PD-L1 expression			p-value
	Negative (<1%)	Medium (1–49%)	High (≥50%)	
**Gender**				0.9213
Female	38 (66.67%)	27 (62.80%)	9 (64.29%)	
Male	19 (33.33%)	16 (37.21%)	5 (35.71%)	
**Age**				0.6615
Age<65	40 (70.18%)	28 (65.12%)	7 (50.00%)	
65≤Age<75	13 (22.81%)	10 (23.26%)	5 (35.71%)	
Age≥75	4 (7.02%)	5 (11.63%)	2 (14.29%)	
**Smoking history**				0.6538
Ever	29 (50.88%)	19 (44.19%)	8 (57.14%)	
Never	28 (49.12%)	24 (55.81%)	6 (42.86%)	
**Prior lines of therapy**				0.5956
0	1 (1.75%)	3 (6.98%)	1 (7.14%)	
1–2	34 (59.65%)	27 (62.79%)	7 (50.00%)	
≥3	22 (38.60%)	13 (30.23%)	6 (42.86%)	
**EGFR status**				0.1711
Major	41 (73.21%)	27 (62.79%)	8 (61.54%)	
Rare	7 (12.50%)	10 (23.26%)	5 (38.46%)	
Others	8 (14.29%)	6 (13.95%)	0 (0.00%)	
**T790M status**				0.0825
Positive	21 (38.18%)	13 (30.95%)	1 (7.14%)	
Negative	34 (61.82%)	29 (69.05%)	13 (92.86%)	

**Figure 3:**
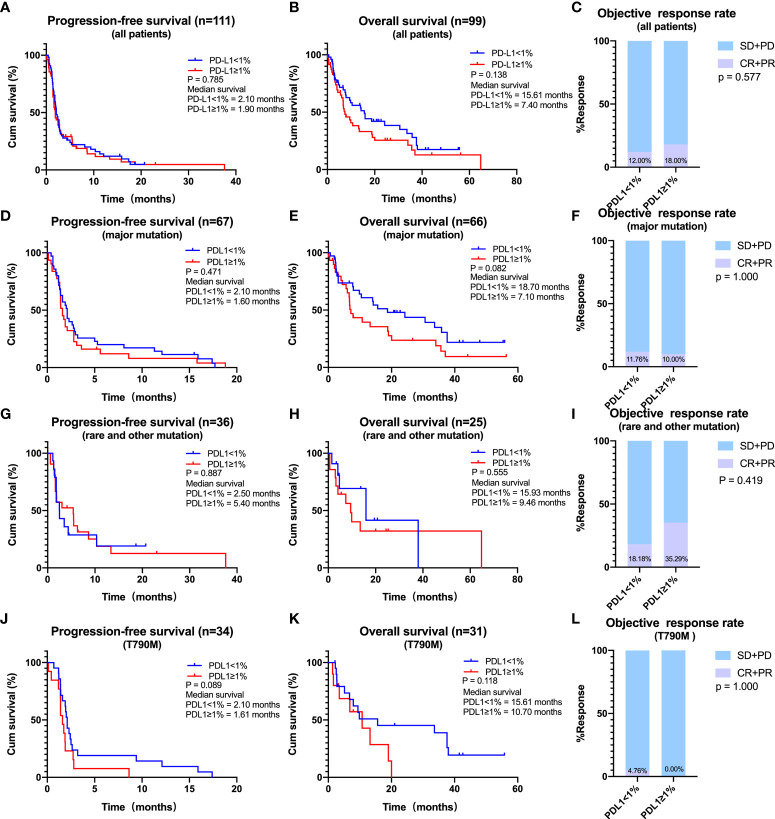
Effects of programmed cell death ligand 1 (PD-L1) expression on the efficacy of immune checkpoint inhibitors (ICIs). **(A–C)** The effect of PD-L1 expression (PD-L1 < 1% *vs*. PD-L1 ≥ 1%) on the progression-free survival (PFS) **(A)**, overall survival (OS) **(B)**, and objective response rate (ORR) **(C)** for all patients receiving ICIs. **(D–F)** The effect of PD-L1 expression (PD-L1 < 1% *vs*. PD-L1 ≥ 1%) on the PFS **(D)**, OS **(E)**, and ORR **(F)** for patients with major mutations receiving ICIs. **(G–I)** The effect of PD-L1 expression (PD-L1 < 1% *vs*. PD-L1 ≥ 1%) on the PFS **(G)**, OS **(H)**, and ORR **(I)** for patients with rare or other mutations receiving ICIs. **(J–L)**, The effect of PD-L1 expression (PD-L1 < 1% *vs*. PD-L1 ≥ 1%) on the PFS **(J)**, OS **(K)**, and ORR **(L)** for patients with T790M receiving ICIs.

In the overall EGFR-mutated population, the median PFS (2.10 vs. 1.90 months, log-rank p = 0.785) was not different between groups of PD-L1 <1% patients and PD-L1 ≥1% patients (**Figure 3A**). The median OS of PD-L1 <1% patients was longer than that of PD-L1 ≥1% patients although not statistically significant. To be specific, the mOS was 15.61 months in PD-L1 <1% patients and 7.40 months in PD-L1 ≥1% patients (log-rank p = 0.138) (**Figure 3B**). ORR (PD-L1 <1% vs. ≥1%; ORR, 12.00% vs. 18.00%, p = 0.577) did not differ (**Figure 3C**). The same tendency was observed in patients with major EGFR mutations, with the median OS being longer in those with PD-L1 negative (<1%) than positive (≥1%) expression (18.70 vs. 7.10 months, log-rank p = 0.082). There was no statistical difference in PFS and ORR between the two subgroups (PD-L1 <1% vs. ≥1%; mPFS, 2.10 vs. 1.60 months, log-rank p = 0.471; ORR, 11.76% vs. 10.00%, p = 1.000) (**Figures 3D–F**). In addition, PD-L1 was not predictive of ICIs efficacy in patients harboring rare or other EGFR mutations (**Figures 3G–I**).

As for patients with EGFR T790M mutations, the most common mechanism for acquired TKIs resistance, the median PFS was longer in patients with PD-L1 <1% compared with those with PD-L1 ≥1% (2.10 vs. 1.61 months, log-rank p = 0.089) (**Figure 3J**). A similar trend was observed for mOS (PD-L1 < 1% vs. ≥ 1%: 15.61 vs. 10.70 months, log-rank p = 0.118) (**Figure 3K**). ORR (PD-L1 <1% vs. ≥1%; ORR, 4.76% vs. 0.00%, p = 1.000) did not differ (**Figure 3L**). However, PFS, OS, and ORR did not differ between patients with low (<50%) and high (≥50%) PD-L1 expression, a finding likely due to the limited number of patients with high PD-L1 expression (**Supplementary Figures 2A–L**).

### Relationships between patients’ clinical characteristics and the efficacies of ICIs

The influence of clinical characteristics, including gender, age, smoking history, and treatment options (monotherapy or combination therapy), on the efficacy of ICIs was evaluated. Univariate and multivariate analyses showed that none of these variables was a risk factor for PFS (all p > 0.05, **Table 3**).

**Table 3:** Univariate and multivariate analyses of each factor’s ability in predicting PFS for 114 EGFR-mutated patients receiving ICIs.

Variables	Univariate analysis		Multivariate analysis	
	OR (95% CI)	p	OR (95% CI)	p
**Gender**		0.752		0.343
Female	Reference		Reference	
Male	1.067 (0.705–1.615)		1.235 (0.798–1.910)	
**Age**		0.578		0.490
Age<65	Reference		Reference	
65≤ Age <75	0.997 (0.623–1.576)	0.968	1.043 (0.648–1.678)	0.862
Age≥75	0.683 (0.373–1.251)	0.255	0.648 (0.308–1.365)	0.254
**Smoking history**		0.172		0.150
Ever	Reference		Reference	
Never	1.304 (0.882–1.928)		1.359 (0.895–2.065)	
**Treatment**		0.799		0.930
Monotherapy	Reference		Reference	
Combination	1.075 (0.602–1.920)		1.027 (0.572–1.841)	

**Figure 4:**
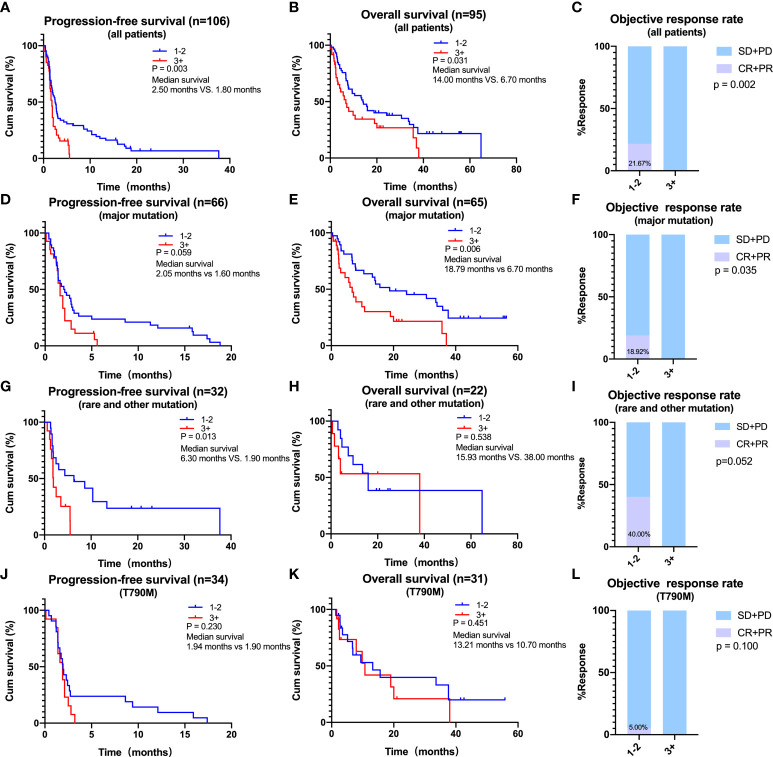
Effects of prior lines of therapy on the efficacy of immune checkpoint inhibitors. **(A–C)** In all patients, progression-free survival (PFS) **(A)**, overall survival (OS) **(B)**, and objective response rate (ORR) **(C)** of patients with 1–2 or ≥ 3 lines of therapy. **(D–F)** In patients harboring major mutations, PFS **(D)**, OS **(E)**, and ORR **(F)** of patients with 1–2 or ≥3 lines of therapy. **(G–I)** In patients harboring rare or other mutations, PFS **(G)**, OS **(H)**, and ORR **(I)** of patients with 1–2 or ≥3 lines of therapy. **(J–L)** In patients harboring T790M, PFS **(J)**, OS **(K)**, and ORR **(L)** of patients with 1–2 or ≥3 lines of therapy.

The effect of prior therapy, an important clinical feature, on the efficacy of ICIs in NSCLC patients with EGFR mutations was also analyzed. As EGFR-TKIs have been the first choice for most patients with EGFR mutations, only 5 (4.4%) of the 114 patients were treatment naive. Thus, we focused on patients who had received treatment before. For all patients, median PFS (2.50 vs. 1.80 months, log-rank p = 0.003) and OS (14.00 vs. 6.70 months, log-rank p = 0.031) were significantly longer in those receiving <3 prior lines of treatment compared to those receiving ≥3 prior lines of treatment (**Figures 4A, B**). ORR was also significantly higher in patients who had received <3 than ≥3 prior lines of treatment (21.67% *vs*. 0.00%, p = 0.002) (**Figure 4C**). Similar findings were observed in patients with major EGFR mutations, with median PFS (2.05 *vs*. 1.60 months, log-rank p = 0.059) and OS (18.79 *vs*. 6.70 months, log-rank p = 0.006) being longer in patients who had received <3 than ≥3 previous lines of treatment (**Figures 4D, E**). ORR was higher in patients who had received <3 than ≥3 prior lines of treatment (18.92% *vs*. 0.00%, p =0.035) (**Figure 4F**). A similar trend was observed in patients with rare mutations and those with T790M mutations, although these differences were not statistically significant (**Figures 4G–L**).

Sequence of treatment is also of importance. To assess the influence of treatment sequence, the effect of ICIs used prior to EGFR-TKIs on clinical outcomes was analyzed. Of the 114 patients in the study cohort, 18 (15.8%) received ICIs before EGFR-TKIs, including 5 (4.4%) who were treatment naive, which meant that they received ICIs as the first-line treatment. Only two of the five treatment-naive patients achieved PR, and both were rare mutations (one with 20ins and the other with G719). Of the 13 other patients who were not treatment naive, 3 achieved PR, harboring 20ins, G719, or L858R mutations, respectively (**Supplementary Table S1**).

## Discussion

Although ICIs have been found to significantly prolong the survival of advanced NSCLC patients harboring wild-type EGFR, their benefits are limited in NSCLC patients with EGFR mutations (21, 22), with some of these patients even developing hyper-progressive disease (HPD) in response to ICIs treatment (23). Some NSCLC patients with EGFR mutations, however, do have response to ICIs, but the characteristics of the potential beneficial population remain obscure. Recognizing that most existing studies included small numbers of patients treated at single center, the present study pooled data from several previous studies and analyzed the characteristics of EGFR-mutated patients who benefited from ICIs.

In agreement with previous results (24–26), the present study found that patients with major EGFR mutations had a poorer response to ICIs than patients with rare EGFR mutations. A possible explanation may be that NSCLC with rare EGFR mutations had higher levels of PD-L1 expression and more abundant CD8+ tumor-infiltrating lymphocytes (TILs) infiltration (27). In addition, research by Dong et al., based on the analysis of the Cancer Genome Atlas cohort and the Guangdong Lung Cancer Institute cohort, showed that tumor mutation burden was significantly lower in patients with treatment-sensitive EGFR mutations than in patients with resistant or unknown EGFR mutations (28). However, when comparing ICIs efficacy on L858R and 19Del, our findings are inconsistent with others. A retrospective study suggested that patients with 19Del mutation have a significantly reduced benefit of treatment with ICIs (20). In our study, there was no statistical difference between L858R and 19Del in terms of PFS, OS, or ORR. An increase in the number of cases may have contributed to the discrepancy.

As for T790M status, our study suggested that T790M positivity was correlated with lower ORR, a finding in agreement with several previous studies. For example, an analysis of 25 patients found that ORR were lower in T790M-positive than in T790M-negative patients (29), and a retrospective study of 24 patients reported that the disease control rate was also lower in T790M-positive patients compared to that in T790M-negative group (25). In addition, these two studies also confirmed that T790M mutations inversely predicted the PFS following treatment with ICIs (25, 29). The IMMUNOTARGET registry study reported similar results (30). One possible reason is that T790M-negative patients had higher levels of PD-L1 expression as compared to T790M-positive patients (29). However, our study showed no statistical difference in PFS and OS between T790M-positive or T790M-negative patients. Another study with 108 patients also found that T790M status had no impact on the benefit from treatment with ICIs (20). Taken together, these controversial findings suggest that sample size can affect these results and that prospective studies involving larger numbers of patients are required to determine the impact of T790M status on the efficacy of ICIs.

To date, immunohistochemical detection of PD-L1 expression on tumor cells has been the most widely used and accepted biomarker for predicting responsiveness to PD-1/PD-L1 inhibitors (31). Multiple clinical trials have proved that higher levels of PD-L1 expression were found to correlate positively with greater benefit from ICIs in patients with advanced NSCLC (32). These results, however, were obtained mainly from patients with wild-type EGFR, with the predictive value of PD-L1 expression in patients with EGFR mutations remaining unclear. Several small retrospective studies have indicated that higher PD-L1 expression in EGFR-mutated patients was associated with longer PFS than lower PD-L1 expression (33, 34). The present study, however, found no association between PD-L1 expression and clinical variables, consistent with previous findings (35). However, contrary to expectations, our results displayed that the overall or major EGFR-mutated patients with higher PD-L1 expression showed a trend towards a shorter OS after receiving ICIs, although the results did not reach statistical significance.

Several possibilities might account for the discrepancy and why EGFR mutant patients with lower PD-L1 expression might have better responses to ICIs. First, the predictive value of PD-L1 as a vital biomarker is affected by its detection methods, including diverse immunohistochemistry platforms and antibodies, variant sample sources (archived or fresh specimens) and handing procedures (timing), and different types of cells assessed (tumor or immune cells). Second and importantly, PD-L1 expression induced by different mechanisms, even at the same level, may contribute to opposite direction of predicting effect. Host anti-tumor immunity is provoked during cancer progression, resulting in the upregulation of PD-L1 by various inflammatory factors, such as IFN-γ, as a negative feedback (36). This “acquired expression” of PD-L1 is a strong indicator of existing immunity, suggesting that ICIs could overcome the immunosuppression by blocking the PD-L1/PD-1 axis and will bring more benefit for this subgroup of patients. In contrast, in EGFR-mutated NSCLC, PD-L1 can be constructively upregulated by EGFR activation and its downstream signaling pathways, such as JAK/STAT/Ras/RAF/MEK/ERK/PI3K/AKT/mTOR (37). This “intrinsic expression” of PD-L1 does not necessarily correlate with pre-existing immune responses, such that patients with tumors positive for PD-L1 are commonly resistant to ICIs (38). The study by Gao et al. showed that overexpression of PD-1 or PD-L1 in tumor cells inhibited tumor cell proliferation, whereas blocking PD-1 or PD-L1 promoted tumor growth *in vitro* and *in vivo* (39). Thus, in the context of absent adaptive immunity, intrinsic PD-L1 expression in tumor cells could decrease tumor progression. In EGFR-mutant NSCLC, tumor microenvironment (TME) tends to be a poorly immunogenic “immune desert” phenotype (40), and thus, it could be speculated that blocking PD-1/PD-L1 would enhance tumor growth and lead to resistance to ICIs, even HPD. HPD has been reported in approximately 20% of patients with EGFR mutations, which is much higher than that in wild-type patients (41). Several studies demonstrated that PD-L1 expression on immune cells were less affected by tumor cell intrinsic factors, such as EGFR activation, and might be a better biomarker. Third, current results were all obtained from retrospective studies. Further prospective research is needed to determine the exact role of PD-L1 in predicting ICIs efficacy in EGFR-mutated NSCLC.

Besides PD-L1, more parameters, particularly factors reflecting features of TME, are emerging as novel biomarkers for predicting the efficacies of ICIs (42). The TME is not only essential for tumor survival and development but also critical for responses to immunotherapeutic strategies (43). EGFR mutations affect multiple components of the TME, and studies have revealed that activation of the EGFR signaling pathway leads to alterations of TME status, including infiltration of immune cells and expression of immunoregulatory cytokines or exosomes (37). For example, EGFR-mutant tumors tend to have high expression of Tregs and CD73 and low infiltration of CD8+ T cells, indicating an immunosuppressive TME (44). Thus, EGFR-mutant NSCLC may have a distinct TME and identifying key factors involved in anti-tumor responses will provide powerful predictive biomarkers for immunotherapy (45, 46). Using high-plex and high-throughput technologies to discover protein biomarkers and molecular phenotypes of NSCLC biopsy samples, a recent study has generated new methodologies for assessing the TME profiles (47).

Treatment strategies and sequence of therapies are crucial to the efficacy of ICIs in EGFR-mutated patients as well. The results of the present study, along with the results of previous studies, suggest that ICIs should be used earlier during the course of treatment. For example, the KEYNOTE-001 trial found that ICIs were more effective in treatment-naive than in previously treated patients, with ORRs of 41.6% and 22.9%, respectively, and median OS of 22.3 and 10.5 months, respectively (48). Moreover, in the KEYNOTE-024 and KEYNOTE-189 trials, the median PFS2, defined as the time from randomization to disease progression after initiation of new anticancer therapy or death from any cause, was longer in patients initially randomized to pembrolizumab or pembrolizumab plus pemetrexed–platinum group, suggesting that ICIs in first-line settings had a greater survival benefit for EGFR-wild type patients (49, 50).

Although this study would provide some clues for the application of ICIs in EGFR-mutated NSCLC patients, yet it had several limitations. First, it was a retrospective analysis of publicly available data rather than a prospective study. Second, although we have collected a relatively large number of EGFR-mutated patients with high PD-L1 expression, it was still not enough for subgroup analysis. Third, this study included patients involved in clinical trials and standard treatments. Thus, their characteristics were not uniform across the groups, which may have led to selection bias. Moreover, this study only discussed the efficacy of ICIs alone in EGFR-mutated NSCLC patients.

## Conclusion and future directions for ICI-based treatment in EGFR-mutated NSCLC

Till now, the modality of treatments for EGFR-mutated advanced NSCLC patients is still controversial. On the basis of the latest National Comprehensive Cancer Network guideline, EGFR-TKIs are the preferred first-line treatment option regardless of PD-L1 expression level (51). Large numbers of studies have demonstrated the superior efficacy of EGFR-TKIs as first-line choice in EGFR-mutated patients. By contrast, ICIs used before EGFR-TKIs showed poor therapeutic efficacy and relatively high toxicity. A phase II trial (http://clinicaltrials.gov/show/NCT02879994) of pembrolizumab in TKI-naive patients with EGFR mutations was ceased because none of these patients responded to pembrolizumab, although 73% of patients had high PD-L1 expression (≥50%) (15). Our study also confirmed that ICIs as first-line treatment showed poor efficacies, especially in patients with major EGFR mutations. Meanwhile, severe immune-related adverse events (irAEs) were observed in patients who were treated with sequential PD-1/PD-L1 inhibitors followed by osimeritinib, but no irAEs were observed when osimertinib preceded PD-(L)1 blockade (52). Besides, combinations of ICIs and EGFR-TKIs are not considered ideal first-line treatments, with many clinical trials halted because of immune-related toxicity issues and limited efficacy (53). Therefore, neither treatment guidelines nor clinical practice considers ICIs as the first-line treatment option for EGFR-mutated patients; rather, EGFR-TKIs remain the first-line treatment of choice for these patients.

However, ICIs should still be used for the treatment of EGFR-mutated NSCLC patients despite their poor first-line treatment efficacy. The results of a real-world study presented at 2021 World Conference on Lung Cancer (WCLC) revealed that patients who received ICIs at any point had longer OS than those who did not receive ICIs, indicating that ICIs still play an important role in the treatment of EGFR-mutated NSCLC patients (54). Moreover, patients who progress on EGFR-TKIs should be started on ICIs as soon as possible. Prospective studies have indicated ICI-based combination therapies would have a better prospect in EGFR-mutated patients, and some studies have shown promising results. For example, Deng’s study has proved that in patients with EGFR-TKI-resistant advanced NSCLC, ICIs plus chemotherapy provided promising ORR and PFS benefit, along with a low rate of severe AEs (55). One study presented at the 2021 WCLC also showed that combined pembrolizumab and chemotherapy improved the response rate of TKIs refractory EGFR-mutated NSCLC patients (56). More promisingly, the IMPOWER 150 uncovered the importance of anti-angiogenic therapy in EGFR-mutated NSCLC. In patients with sensitizing EGFR mutations, improved OS was observed in atezolizumab plus bevacizumab plus carboplatin plus paclitaxel (ABCP) group rather than bevacizumab plus carboplatin plus paclitaxel (BCP) group (57). The ORIENT-31 trial has also demonstrated that treatment with anti-PD-1 and anti-angiogenesis plus chemotherapy significantly improved PFS in patients with EGFR-mutated non-squamous NSCLC resistant to EGFR-TKIs (58).

**Figure 5:**
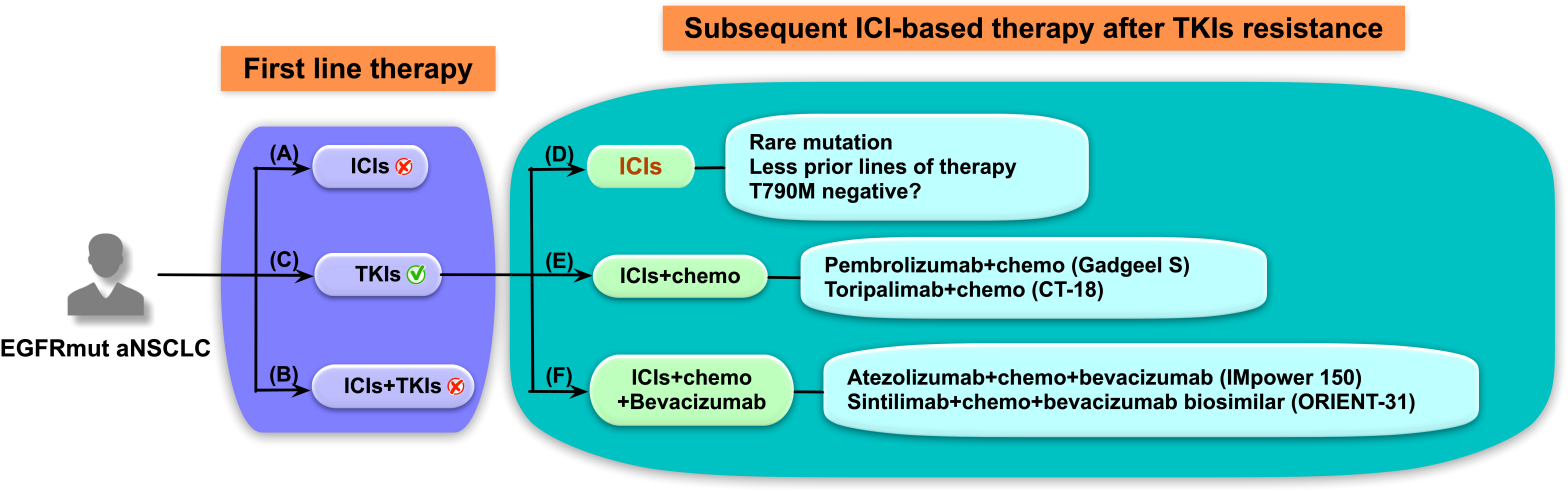
Schematic diagram of treatment strategies for advanced non-small cell lung cancer (NSCLC) patients with epidermal growth factor receptor (EGFR) mutations. Immune checkpoint inhibitor (ICI)-based treatment strategies for EGFR-mutated (EGFRmut) patients with advanced NSCLC should be elaborately designed. **(A–C)** In the first-line setting, **(A)** ICIs was found to lack efficacy and subsequent EGFR-tyrosine kinase inhibitors (TKIs) treatment possibly leads to severe immune-related adverse events, and **(B)** ICIs in combination with TKIs also resulted in high treatment-related toxicities and poor tolerance. **(C)** EGFR-TKIs are still preferred for the first-line therapy of EGFRmut patients regardless of programmed cell death ligand 1 expression. **(D–F)** As for subsequent ICI-based therapy after TKIs resistance, **(D)** ICIs alone may have better efficacy in EGFRmut patients with rare mutation or patients who had received fewer prior lines of therapy. ICI-based combined therapies, including **(E)** ICIs plus chemotherapy and **(F)** ICIs plus chemotherapy and anti-angiogenic therapy, have shown promising results in prospective clinical studies and represent the future treatment strategies and direction for these patients.

In conclusion (**Figure 5**), EGFR-TKIs are still the preferred first-line treatment for EGFR-mutant NSCLC. After TKIs resistance, ICI-based combination therapies are the direction for future treatment. However, not all patients could tolerate combination therapy and ICIs alone is a choice for EGFR-mutant patients with beneficial features. Moreover, as it is difficult to identify the dominant subgroups that will benefit from ICI-based combination therapies, our findings of ICI therapy alone from this study may provide some clues.

## Data availability statement

The original contributions presented in the study are included in the article/supplementary material. Further inquiries can be directed to the corresponding author/s.

## Author contributions

QC, XS, NL, XM, WH, XW and YL conceived and designed this work. QC, XS and YL analyzed the data. QC and YL wrote the paper. All authors contributed to the article and approved the submitted version.

## Funding

This work was supported by grants from the National Natural Science Foundation of China (No. 81874044) and the Shandong Provincial Natural Science Foundation (Nos. ZR2020MH236 and ZR2019MH050).

## Conflict of interest

The authors declare that the research was conducted in the absence of any commercial or financial relationships that could be construed as a potential conflict of interest.

## Publisher’s note

All claims expressed in this article are solely those of the authors and do not necessarily represent those of their affiliated organizations, or those of the publisher, the editors and the reviewers. Any product that may be evaluated in this article, or claim that may be made by its manufacturer, is not guaranteed or endorsed by the publisher.

## References

[1] Sung H Ferlay J Siegel RL Laversanne M Soerjomataram I Jemal A. Global cancer statistics 2020: GLOBOCAN estimates of incidence and mortality worldwide for 36 cancers in 185 countries. CA Cancer J Clin (2021) 71:209–49. doi: 10.3322/caac.21660 33538338

[2] Cao W Chen HD Yu YW Li N Chen WQ. Changing profiles of cancer burden worldwide and in China: a secondary analysis of the global cancer statistics 2020. Chin Med J (Engl) (2021) 134:783–9. doi: 10.1097/cm9.0000000000001474 PMC8104205 33734139

[3] Shi Y Au JS Thongprasert S Srinivasan S Tsai CM Khoa MT. A prospective, molecular epidemiology study of EGFR mutations in Asian patients with advanced non-small-cell lung cancer of adenocarcinoma histology (PIONEER). J Thorac Oncol (2014) 9:154–62. doi: 10.1097/jto.0000000000000033 PMC4132036 24419411

[4] Mok TS Wu YL Thongprasert S Yang CH Chu DT Saijo N. Gefitinib or carboplatin-paclitaxel in pulmonary adenocarcinoma. N Engl J Med (2009) 361:947–57. doi: 10.1056/NEJMoa0810699 19692680

[5] Sequist LV Yang JC Yamamoto N O'Byrne K Hirsh V Mok T. Phase III study of afatinib or cisplatin plus pemetrexed in patients with metastatic lung adenocarcinoma with EGFR mutations. J Clin Oncol (2013) 31:3327–34. doi: 10.1200/jco.2012.44.2806 23816960

[6] Soria JC Ohe Y Vansteenkiste J Reungwetwattana T Chewaskulyong B Lee KH. Osimertinib in untreated EGFR-mutated advanced non-Small-Cell lung cancer. N Engl J Med (2018) 378:113–25. doi: 10.1056/NEJMoa1713137 29151359

[7] O'Leary C Gasper H Sahin KB Tang M Kulasinghe A Adams MN. Epidermal growth factor receptor (EGFR)-mutated non-Small-Cell lung cancer (NSCLC). Pharm (Basel) (2020) 13:273. doi: 10.3390/ph13100273 PMC7600164 32992872

[8] Passaro A Jänne PA Mok T Peters S. Overcoming therapy resistance in EGFR-mutant lung cancer. Nat Cancer (2021) 2:377–91. doi: 10.1038/s43018-021-00195-8 35122001

[9] Borghaei H Paz-Ares L Horn L Spigel DR Steins M Ready NE. Nivolumab versus docetaxel in advanced nonsquamous non-Small-Cell lung cancer. N Engl J Med (2015) 373:1627-39. doi: 10.1056/NEJMoa1507643 PMC5705936 26412456

[10] Brahmer J Reckamp KL Baas P Crinò L Eberhardt WE Poddubskaya E. Nivolumab versus docetaxel in advanced squamous-cell non-Small-Cell lung cancer. N Engl J Med (2015) 373:123-35. doi: 10.1056/NEJMoa1504627 PMC4681400 26028407

[11] Fehrenbacher L Spira A Ballinger M Kowanetz M Vansteenkiste J Mazieres J. Atezolizumab versus docetaxel for patients with previously treated non-small-cell lung cancer (POPLAR): a multicentre, open-label, phase 2 randomised controlled trial. Lancet (2016) 387:1837-46. doi: 10.1016/s0140-6736(16)00587-0 26970723

[12] Herbst RS Baas P Kim DW Felip E Pérez-Gracia JL Han JY. Pembrolizumab versus docetaxel for previously treated, PD-L1-positive, advanced non-small-cell lung cancer (KEYNOTE-010): a randomised controlled trial. Lancet (2016) 387:1540–50. doi: 10.1016/s0140-6736(15)01281-7 26712084

[13] Rittmeyer A Barlesi F Waterkamp D Park K Ciardiello F von Pawel J. Atezolizumab versus docetaxel in patients with previously treated non-small-cell lung cancer (OAK): a phase 3, open-label, multicentre randomised controlled trial. Lancet (2017) 389:255–65. doi: 10.1016/s0140-6736(16)32517-x PMC6886121 27979383

[14] Gettinger S Rizvi NA Chow LQ Borghaei H Brahmer J Ready N. Nivolumab monotherapy for first-line treatment of advanced non-Small-Cell lung cancer. J Clin Oncol (2016) 34:2980–7. doi: 10.1200/JCO.2016.66.9929 PMC5569692 27354485

[15] Lisberg A Cummings A Goldman JW Bornazyan K Reese N Wang T. A phase II study of pembrolizumab in EGFR-mutant, PD-L1+, tyrosine kinase inhibitor naïve patients with advanced NSCLC. J Thorac Oncol (2018) 13:1138–45. doi: 10.1016/j.jtho.2018.03.035 PMC6063769 29874546

[16] Garassino MC Cho BC Kim JH Mazières J Vansteenkiste J Lena H. Final overall survival and safety update for durvalumab in third- or later-line advanced NSCLC: The phase II ATLANTIC study. Lung Cancer (2020) 147:137–42. doi: 10.1016/j.lungcan.2020.06.032 32702570

[17] Garassino MC Cho BC Kim JH Mazières J Vansteenkiste J Lena H. Durvalumab as third-line or later treatment for advanced non-small-cell lung cancer (ATLANTIC): an open-label, single-arm, phase 2 study. Lancet Oncol (2018) 19:521-36. doi: 10.1016/s1470-2045(18)30144-x PMC7771363 29545095

[18] Gandara DR Paul SM Kowanetz M Schleifman E Zou W Li Y. Blood-based tumor mutational burden as a predictor of clinical benefit in non-small-cell lung cancer patients treated with atezolizumab. Nat Med (2018) 24:1441–8. doi: 10.1038/s41591-018-0134-3 30082870

[19] Rizvi H Sanchez-Vega F La K Chatila W Jonsson P Halpenny D. Molecular determinants of response to anti-programmed cell death (PD)-1 and anti-programmed death-ligand 1 (PD-L1) blockade in patients with non-Small-Cell lung cancer profiled with targeted next-generation sequencing. J Clin Oncol (2018) 36:633–41. doi: 10.1200/JCO.2017.75.3384 PMC6075848 29337640

[20] Hastings K Yu HA Wei W Sanchez-Vega F DeVeaux M Choi J. EGFR mutation subtypes and response to immune checkpoint blockade treatment in non-small-cell lung cancer. Ann Oncol (2019) 30:1311–20. doi: 10.1093/annonc/mdz141 PMC6683857 31086949

[21] Gainor JF Shaw AT Sequist LV Fu X Azzoli CG Piotrowska Z. ) EGFR mutations and ALK rearrangements are associated with low response rates to PD-1 pathway blockade in non-small cell lung cancer: A retrospective analysis. Clin Cancer Res (2016) 22:4585–93. doi: 10.1158/1078-0432.Ccr-15-3101 PMC5026567 27225694

[22] Lee CK Man J Lord S Links M Gebski V Mok T. Checkpoint inhibitors in metastatic EGFR-mutated non-small cell lung cancer-a meta-analysis. J Thorac Oncol (2017) 12:403–7. doi: 10.1016/j.jtho.2016.10.007 27765535

[23] Oguri T Sasada S Seki S Murata S Tsuchiya Y Ishioka K. A case of hyperprogressive disease following atezolizumab therapy for pulmonary pleomorphic carcinoma with epidermal growth factor receptor mutation. Respir Med Case Rep (2021) 33:101405. doi: 10.1016/j.rmcr.2021.101405 33868915 PMC8042444

[24] Yoshida H Kim YH Ozasa H Nagai H Sakamori Y Tsuji T. Nivolumab in non-small-cell lung cancer with EGFR mutation. Ann Oncol (2018) 29. doi: 10.1093/annonc/mdx745 29161357

[25] Yamada T Hirai S Katayama Y Yoshimura A Shiotsu S Watanabe S. Retrospective efficacy analysis of immune checkpoint inhibitors in patients with EGFR-mutated non-small cell lung cancer. Cancer Med (2019) 8:1521–9. doi: 10.1002/cam4.2037 PMC6488155 30790471

[26] Lau SCM Fares AF Le LW Mackay KM Soberano S Chan SW. Subtypes of EGFR- and HER2-mutant metastatic NSCLC influence response to immune checkpoint inhibitors. Clin Lung Cancer (2021) 22:253–9. doi: 10.1016/j.cllc.2020.12.015 33582070

[27] Chen K Cheng G Zhang F Zhu G Xu Y Yu X. PD-L1 expression and T cells infiltration in patients with uncommon EGFR-mutant non-small cell lung cancer and the response to immunotherapy. Lung Cancer (2020) 142:98–105. doi: 10.1016/j.lungcan.2020.02.010 32120230

[28] Dong ZY Zhang JT Liu SY Su J Zhang C Xie Z. EGFR mutation correlates with uninflamed phenotype and weak immunogenicity, causing impaired response to PD-1 blockade in non-small cell lung cancer. Oncoimmunology (2017) 6. doi: 10.1080/2162402x.2017.1356145 PMC5674946 29147605

[29] Haratani K Hayashi H Tanaka T Kaneda H Togashi Y Sakai K. Tumor immune microenvironment and nivolumab efficacy in EGFR mutation-positive non-small-cell lung cancer based on T790M status after disease progression during EGFR-TKI treatment. Ann Oncol (2017) 28::1532–9. doi: 10.1093/annonc/mdx183 28407039

[30] Mazieres J Drilon A Lusque A Mhanna L Cortot AB Mezquita L. Immune checkpoint inhibitors for patients with advanced lung cancer and oncogenic driver alterations: results from the IMMUNOTARGET registry. Ann Oncol (2019) 30:1321–8. doi: 10.1093/annonc/mdz167 PMC7389252 31125062

[31] Teixido C Vilarino N Reyes R Reguart N. PD-L1 expression testing in non-small cell lung cancer. Ther Adv Med Oncol (2018) 10:1758835918763493. doi: 10.1177/1758835918763493 29662547 PMC5898658

[32] Bodor JN Boumber Y Borghaei H. Biomarkers for immune checkpoint inhibition in non-small cell lung cancer (NSCLC). Cancer (2020) 126:260–70. doi: 10.1002/cncr.32468 PMC7372560 31691957

[33] Ichihara E Harada D Inoue K Shibayama T Hosokawa S Kishino D. Characteristics of patients with EGFR-mutant non-small-cell lung cancer who benefited from immune checkpoint inhibitors. Cancer Immunol Immunother (2021) 70:101–6. doi: 10.1007/s00262-020-02662-0 PMC10991645 32648165

[34] Masuda K Horinouchi H Tanaka M Higashiyama R Shinno Y Sato J. Efficacy of anti-PD-1 antibodies in NSCLC patients with an EGFR mutation and high PD-L1 expression. J Cancer Res Clin Oncol (2021) 147(1):245–51. doi: 10.1007/s00432-020-03329-0 PMC7810613 32705363

[35] Brody R Zhang Y Ballas M Siddiqui MK Gupta P Barker C. PD-L1 expression in advanced NSCLC: Insights into risk stratification and treatment selection from a systematic literature review. Lung Cancer (2017) 112:200–15. doi: 10.1016/j.lungcan.2017.08.005 29191596

[36] Mandai M Hamanishi J Abiko K Matsumura N Baba T Konishi I. Dual faces of IFNgamma in cancer progression: A role of PD-L1 induction in the determination of pro- and antitumor immunity. Clin Cancer Res (2016) 22:2329–34. doi: 10.1158/1078-0432.CCR-16-0224 27016309

[37] Lin A Wei T Meng H Luo P Zhang J. Role of the dynamic tumor microenvironment in controversies regarding immune checkpoint inhibitors for the treatment of non-small cell lung cancer (NSCLC) with EGFR mutations. Mol Cancer (2019) 18(1):139. doi: 10.1186/s12943-019-1062-7 31526368 PMC6745797

[38] Yi M Niu M Xu L Luo S Wu K. Regulation of PD-L1 expression in the tumor microenvironment. J Hematol Oncol (2021) 14(1):10. doi: 10.1186/s13045-020-01027-5 33413496 PMC7792099

[39] Wang X Yang X Zhang C Wang Y Cheng T Duan L. Tumor cell-intrinsic PD-1 receptor is a tumor suppressor and mediates resistance to PD-1 blockade therapy. Proc Natl Acad Sci U.S.A. (2020) 117:6640–50. doi: 10.1073/pnas.1921445117 PMC7104341 32161124

[40] Budczies J Kirchner M Kluck K Kazdal D Glade J Allgauer M. Deciphering the immunosuppressive tumor microenvironment in ALK- and EGFR-positive lung adenocarcinoma. Cancer Immunol Immunother (2022) 71:251–65. doi: 10.1007/s00262-021-02981-w PMC8783861 34125345

[41] To KKW Fong W Cho WCS. Immunotherapy in treating EGFR-mutant lung cancer: Current challenges and new strategies. Front Oncol (2021) 11:635007. doi: 10.3389/fonc.2021.635007 34113560 PMC8185359

[42] Sadeghi Rad H Monkman J Warkiani ME Ladwa R O'Byrne K Rezaei N. Understanding the tumor microenvironment for effective immunotherapy. Med Res Rev (2021) 41:1474–98. doi: 10.1002/med.21765 PMC8247330 33277742

[43] Bejarano L Jordāo MJC Joyce JA. Therapeutic targeting of the tumor microenvironment. Cancer Discovery (2021) 11:933–59. doi: 10.1158/2159-8290.Cd-20-1808 33811125

[44] Ma L Diao B Huang Z Wang B Yu J Meng X. The efficacy and possible mechanisms of immune checkpoint inhibitors in treating non-small cell lung cancer patients with epidermal growth factor receptor mutation. Cancer Commun (Lond) (2021) 41:1314–30. doi: 10.1002/cac2.12229 PMC8696228 34699691

[45] Lei Y Wang K Liu Y Wang X Xiang X Ning X. Various subtypes of EGFR mutations in patients with NSCLC define genetic, immunologic diversity and possess different prognostic biomarkers. Front Immunol (2022) 13:811601. doi: 10.3389/fimmu.2022.811601 35265073 PMC8899028

[46] Santaniello A Napolitano F Servetto A De Placido P Silvestris N Bianco C. Tumour microenvironment and immune evasion in EGFR addicted NSCLC: Hurdles and possibilities. Cancers (Basel) (2019) 11(10):1419. doi: 10.3390/cancers11101419 PMC6826622 31554160

[47] Monkman J Taheri T Ebrahimi Warkiani M O'Leary C Ladwa R Richard D. High-plex and high-throughput digital spatial profiling of non-Small-Cell lung cancer (NSCLC). Cancers (Basel) (2020) 12(12):3551. doi: 10.3390/cancers12123551 PMC7760230 33261133

[48] Garon EB Hellmann MD Rizvi NA Carcereny E Leighl NB Ahn MJ. Five-year overall survival for patients with advanced Non-Small-cell lung cancer treated with pembrolizumab: Results from the phase I KEYNOTE-001 study. J Clin Oncol (2019) 37:2518–27. doi: 10.1200/jco.19.00934 PMC6768611 31154919

[49] Rodriguez-Abreu D Powell SF Hochmair MJ Gadgeel S Esteban E Felip E. Pemetrexed plus platinum with or without pembrolizumab in patients with previously untreated metastatic nonsquamous NSCLC: protocol-specified final analysis from KEYNOTE-189. Ann Oncol (2021) 32:881–95. doi: 10.1016/j.annonc.2021.04.008 33894335

[50] Reck M Rodríguez-Abreu D Robinson AG Hui R Csőszi T Fülöp A. Five-year outcomes with pembrolizumab versus chemotherapy for metastatic non-Small-Cell lung cancer with PD-L1 tumor proportion score ≥ 50. J Clin Oncol (2021) 39:2339–49. doi: 10.1200/jco.21.00174 PMC8280089 33872070

[51] Ettinger DS Wood DE Aisner DL NCCN clinical practice guidelines in oncology (NCCN guidelines) non-small cell lung cancer. NCCN (2021). Available at: www.nccn.org/patients.

[52] Schoenfeld AJ Arbour KC Rizvi H Iqbal AN Gadgeel SM Girshman J. Severe immune-related adverse events are common with sequential PD-(L)1 blockade and osimertinib. Ann Oncol (2019) 30:839–44. doi: 10.1093/annonc/mdz077 PMC7360149 30847464

[53] Qiao M Jiang T Liu X Mao S Zhou F Li X. Immune checkpoint inhibitors in EGFR-mutated NSCLC: Dusk or dawn? J Thorac Oncol (2021) 16::1267–88. doi: 10.1016/j.jtho.2021.04.003 33915248

[54] Ali F Zhang C Khaddour L Gadgeel SM Tadesse E Thompson MA. P10.04-immunotherapy-treated non-small cell lung cancer patients with sensitizing gene alterations: a real world survival analysis. World Conf Lung Cancer (2021) 16(10):S999–1000. doi: 10.1016/j.jtho.2021.08.309.

[55] Deng H Lin X Xie X Yang Y Wang L Wu J. Immune checkpoint inhibitors plus single-agent chemotherapy for advanced non-Small-Cell lung cancer after resistance to EGFR-TKI. Front Oncol (2021) 11:700023. doi: 10.3389/fonc.2021.700023 34616674 PMC8488293

[56] Nagasaka M Dziubek K Gadgeel SM Braun T Hassan K Cheng H. OA09.03-pembrolizumab in combination with platinumbased chemotherapy in recurrent EGFR/ALK+ non-small cell lung cancer (NSCLC). World Conf Lung Cancer (2021) 16(10):S863. doi: 10.1016/j.jtho.2021.08.063.

[57] Reck M Mok TSK Nishio M Jotte RM Cappuzzo F Orlandi F. Atezolizumab plus bevacizumab and chemotherapy in non-small-cell lung cancer (IMpower150): key subgroup analyses of patients with EGFR mutations or baseline liver metastases in a randomised, open-label phase 3 trial. Lancet Respir Med (2019) 7:387–401. doi: 10.1016/S2213-2600(19)30084-0 30922878

[58] Lu S Wu L Jian H Pan Y Ye F Liu A. Orient-31: Phase iii study of sintilimab with or without Ibi305 plus chemotherapy in patients with egfr mutated nonsquamous nsclc who progressed after egfr-tki therapy. ESMO Asia Virtual Oncol Week (2021) 33(1):P112–3. doi: 10.1016/j.annonc.2021.10.007.

