# Supplementary material for: Features of patients with advanced EGFR-mutated non-small cell lung cancer benefiting from immune checkpoint inhibitors
Source: Front Immunol. 2022 Aug 5;13:931718. doi: 10.3389/fimmu.2022.931718 (PMC9388930; doi:10.3389/fimmu.2022.931718)
Supplement: Supplementary file 1 [file DataSheet_1.docx]

Supplementary Material

# Supplementary Figures and Tables

## Supplementary Figures


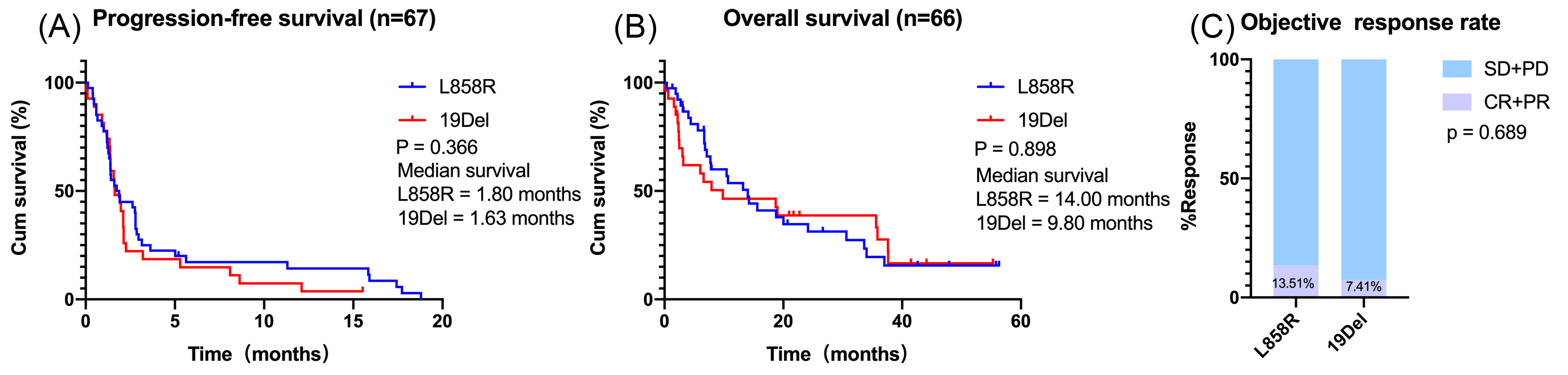


**Supplementary Figure 1.** Efficacy of immune checkpoint inhibitors on distinct major epidermal growth factor receptor (EGFR) subtypes. Progression-free survival (A), overall survival (B), and objective response rate (C) in patients harboring EGFR L858R and 19Del mutations.


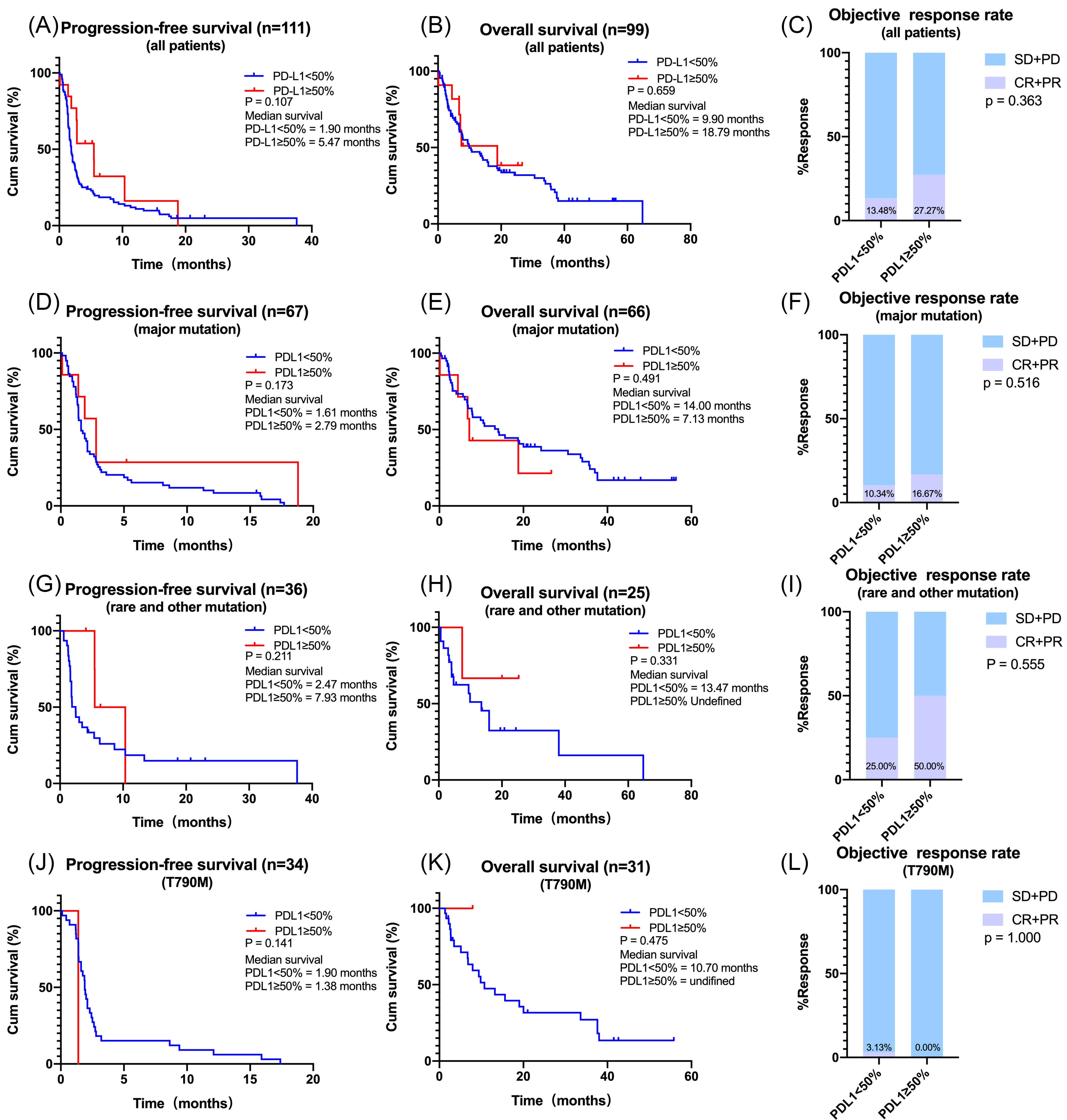


**Supplementary Figure 2.** Effect of programmed cell death ligand 1 (PD-L1) expression on the efficacy of immune checkpoint inhibitors (ICIs). (A-C), The effect of PD-L1 expression (PD-L1 < 50% vs. PD-L1 ≥ 50%) on the progression-free survival (PFS) (A), overall survival (OS) (B), and objective response rate (ORR) (C) for all patients receiving ICIs. (D-F), The effect of PD-L1 expression (PD-L1 < 50% vs. PD-L1 ≥ 50%) on the PFS (D), OS (E), and ORR (F) for patients with major mutations receiving ICIs. (G-I), The effect of PD-L1 expression (PD-L1 < 50% vs. PD-L1 ≥ 50%) on the PFS (G), OS (H), and ORR (I) for patients with rare or other mutations receiving ICIs. (J-L), The effect of PD-L1 expression (PD-L1 < 50% vs. PD-L1 ≥ 50%) on the PFS (J), OS (K), and ORR (L) for patients with T790M receiving ICIs.

## Supplementary Tables

| **Supplementary Table 1. Characteristics of 18 EGFR-TKI naive patients** | | | | |
| --- | --- | --- | --- | --- |
| **Study ID** | **TKI prior to start of ICI therapy?** | **EGFR Mutation** | **Prior lines of therapy** | **Best Response** |
| 9833 | no | 20ins | 0 | PD |
| Patient 16 | no | 20ins | 0 | PD |
| Patient 32 | no | 20ins | 0 | PR |
| Patient 36 | no | Del19 | 0 | SD |
| EGFR-IO 35 | no | G719 | 0 | PR |
| 9390 | no | 20ins | 1-2 | PR |
| EGFR-IO 34 | no | 20ins | 1-2 | PD |
| Patient 14 | no | 20ins | 1-2 | PD |
| IN_002 | no | Del19 | 1-2 | SD |
| Patient 22 | no | Del19 | 1-2 | PD |
| EGFR-IO 28 | no | G719 | 1-2 | SD |
| Patient 30 | no | G719 | 1-2 | PR |
| 8858 | no | L858R | 1-2 | PR |
| CN_366 | no | L858R | 1-2 | PD |
| IN_006 | no | L858R | 1-2 | SD |
| CN_310 | no | Other | 1-2 | PD |
| CN_141 | no | 20ins | 3+ | PD |
| CN_142 | no | 20ins | 3+ | PD |
